# Seasonal Migration Determined by a Trade-Off between Predator Avoidance and Growth

**DOI:** 10.1371/journal.pone.0001957

**Published:** 2008-04-16

**Authors:** Christer Brönmark, Christian Skov, Jakob Brodersen, P. Anders Nilsson, Lars-Anders Hansson

**Affiliations:** 1 Department of Ecology, Limnology, Lund University, Lund, Sweden; 2 Danish Institute for Fisheries Research, Silkeborg, Denmark; University of Pretoria, South Africa

## Abstract

Migration is a common phenomenon in many organisms, terrestrial as well as aquatic, and considerable effort has been spent to understand the evolution of migratory behaviour and its consequences for population and community dynamics. In aquatic systems, studies on migration have mainly been focused on commercially important fish species, such as salmon and trout. However, seasonal mass-migrations may occur also among other freshwater fish, e.g. in cyprinids that leave lakes and migrate into streams and wetlands in the fall and return back to the lake in spring. In a conceptual model, we hypothesized that this is an adaptive behaviour in response to seasonal changes in predation (P) and growth (G) and that migrating fish change habitat so as to minimise the ratio between predation mortality and growth rate (P/G). Estimates from bioenergetic modelling showed that seasonal changes in the ratio between predator consumption rate and prey growth rate followed the predictions from the conceptual model and also gave more precise predictions for the timing of the habitat change. By quantifying the migration of more than 1800 individually marked fish, we showed that actual migration patterns followed predictions with a remarkable accuracy, suggesting that migration patterns have evolved in response to seasonally fluctuating trade-offs between predator avoidance and foraging gains. Thus, the conceptual model provides a mechanistic understanding to mass–migration in prey fish. Further, we also show that the dominant prey fish is actually absent from the lake during a major part of the year, which should have strong implications for the dynamics of the lake ecosystem through direct and indirect food-web interactions.

## Introduction

Migration is a common phenomenon in many organisms and occurs regularly in all kinds of environments, terrestrial as well as aquatic. It may be defined as a synchronised movement of all or a large part of a population between two or more separate habitats [1]–[3]. The distances moved are large relative to the average home range and migration usually occurs with a regular periodicity at specific stages of a species life-cycle. Animal migration may have strong effects on the population dynamics of the migrating species and, as a consequence, affects species interactions and community structure and function, but also the flux of, for example, nutrients between different systems [4], [5]. Given its importance, a substantial amount of work has been done to understand the evolution of migratory behaviour and its consequences for population and community dynamics [1], [6], [7].

In fish, migration is often associated with the large-scale movements between breeding and feeding grounds, e.g. in salmonids [8]. However, migration in fish may occur at a range of spatial and temporal scales, from diel migration among habitats to seasonal migrations on a landscape level [2]. In streams and rivers, many fish species move from fast-flowing, shallow areas to slower, deeper sections downstream in the autumn or, alternatively, leave the main river channel and move into backwaters and tributaries where they stay during winter [2]. Recent observations from shallow lakes have also suggested that a large proportion of the cyprinid populations leave the lake during winter and move up into streams and wetlands in the watershed [9, Brönmark, Hansson, Skov, personal observations].

Traditionally, the study of migration patterns in fishes has mainly been concerned with describing migration trajectories and the environmental factors that act as proximate cues for migration behaviour. Less focus has been on the ultimate causes behind migration, i.e. the factors involved in the evolution of different migration strategies [8]. However, seasonal migration has often been regarded as an adaptive strategy to increase growth and survival and, thus, to maximize fitness, in seasonally fluctuating environments [3]. Migrating individuals may benefit from increased food availability or by avoiding harsh abiotic or biotic conditions. Predation is a strong mortality factor for fish and numerous studies have shown that a change in predation risk induces behavioural habitat shifts of prey over a range of spatial and temporal scales, including diel migrations between refuge and feeding habitats [10], ontogenetic habitat shifts from the pelagic to the more structurally complex littoral zone in juvenile fish [11]–[13] and migration out of a lake into streams in response to piscivore introduction [14]. Such habitat shifts may often involve trade-offs, for example the trade-off between risk avoidance (predation) and foraging return, and a number of studies have shown that fish are able to trade-off potential costs and benefits of foraging in habitats with different levels of piscivore threat [15]–[18]. Werner and Gilliam [19] provided a theoretical framework for decision rules for habitat choice in organisms exposed to such conflicting demands, specifically ontogenetic habitat shifts in fish. Their model predicts that a juvenile in a size-structured population should choose habitat so that the ratio of instantaneous mortality rate to growth rate is minimized. Thus, foragers may accept the risk of higher predation mortality if the foraging return is high or, alternatively, avoid feeding in habitats with high predation risk. A number of field and laboratory studies on behavioural decisions have supported the model [20] and it has also been suggested that this general framework may be useful in understanding the evolution of migratory behaviour in fish [21]–[25].

In this study, we evaluate the hypothesis that seasonal migration in fish is an adaptation that has evolved in response to seasonal changes in risk of predation (P) and growth (G) and, thus, that migrating fish change habitat so as to minimise the ratio (P/G) between predation mortality and growth rate. To evaluate this hypothesis we first need to understand the seasonal changes in costs and benefits in terms of predation risk and growth rate associated with two habitats, the lake and its streams, and we therefore developed a conceptual model based on the general predictions from Werner and Gilliam [19].

**Figure 1:**
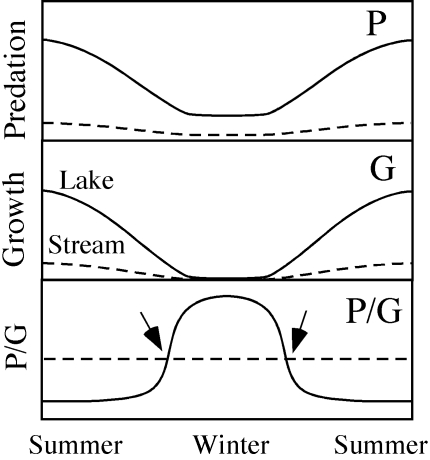
A conceptual model for seasonal changes in predation rate by piscivores, growth rate in zooplanktivorous fish and the trade-off, i.e. the ratio of predation and growth, in the lake and stream habitat. Migrating fish are expected to change habitat so that they minimize the ratio and, thus, migrate from the lake to the stream in autumn and back to the lake in spring, as indicated by arrows.

We assume that seasonal changes in predation pressure and growth are driven by temperature, as temperature is a major determinant of fish foraging and growth rates [26]. However, differences in optimal temperature ranges among predators and prey may create asymmetries in their seasonal consumption rate patterns. In the lake, consumption rates of predators, and thus mortality rates for prey fish, should be highest during summer and decrease to low levels during winter (Fig. 1). However, predatory fish do feed even during cold winter months [27], [28] and, thus, there is a predation risk also during winter. For prey fish, food availability (zooplankton) is highest in the summer, decreasing to low levels during winter [e.g. 29]. In combination with a low temperature threshold for feeding and growth (e.g. 12–15°C in roach [30], [31]), this should result in low growth rates in prey fish in the winter (Fig. 1). Thus, the trade-off, i.e. the ratio (P/G), between predation mortality (P) and growth (G) for prey fish in the lake should be low during summer due to high growth rates (Fig. 1), whereas in winter, even though absolute predation rate is low, the low growth rate should result in a high trade-off ratio (high P/G; Fig. 1). In the stream, we assume that the density of predatory fish, and thus predation pressure, is low all year and, further, that there is a constantly low growth rate for planktivorous fish as the zooplankton food resource is almost absent. This should result in a low and almost constant predation to growth ratio (P/G) in the stream throughout the year (Fig. 1). Thus, we hypothesize that there is a strong selection pressure for adaptive seasonal migration from the lake to the predation refuge in the stream habitat driven by seasonal changes in predation pressure and growth potential in the lake. We predict that roach should leave the lake during autumn and return to the lake in spring when temperature, resource levels and, thus, growth potential increase (Fig. 1). More precisely, prey fish should shift habitat so as to minimize the ratio between predation rate and growth, i.e. at the time when the ratios for respective habitats cross in autumn and spring (Fig. 1).

To test the predictions from the conceptual model we first estimated seasonal patterns in predation (P) and growth (G) using a bioenergetics model, as seasonal changes in predation and growth rates are assumed to be driven mainly by temperature. The bioenergetics model also give more precise predictions on the timing of the habitat shift. Lastly, we tested the predictions empirically by quantifying migration patterns of prey fish over two consecutive seasons in a model system, Lake Krankesjön, in southern Sweden.

## Results and Discussion

In the autumn a large number of the dominant prey fish, roach (*Rutilus rutilus*), migrated from the lake into the streams, where they stayed until spring. Very few predatory pike (*Esox lucius*) and perch (*Perca fluviatilis*) were found in the streams (0.1±0.2% and 0.8±1.2% of total catches, respectively) and, further, the absolute majority of the PIT-tagged predators stayed in the lake during winter. Thus, the empirical data showed a massive migration of the major prey fish into the streams during winter.

**Figure 2:**
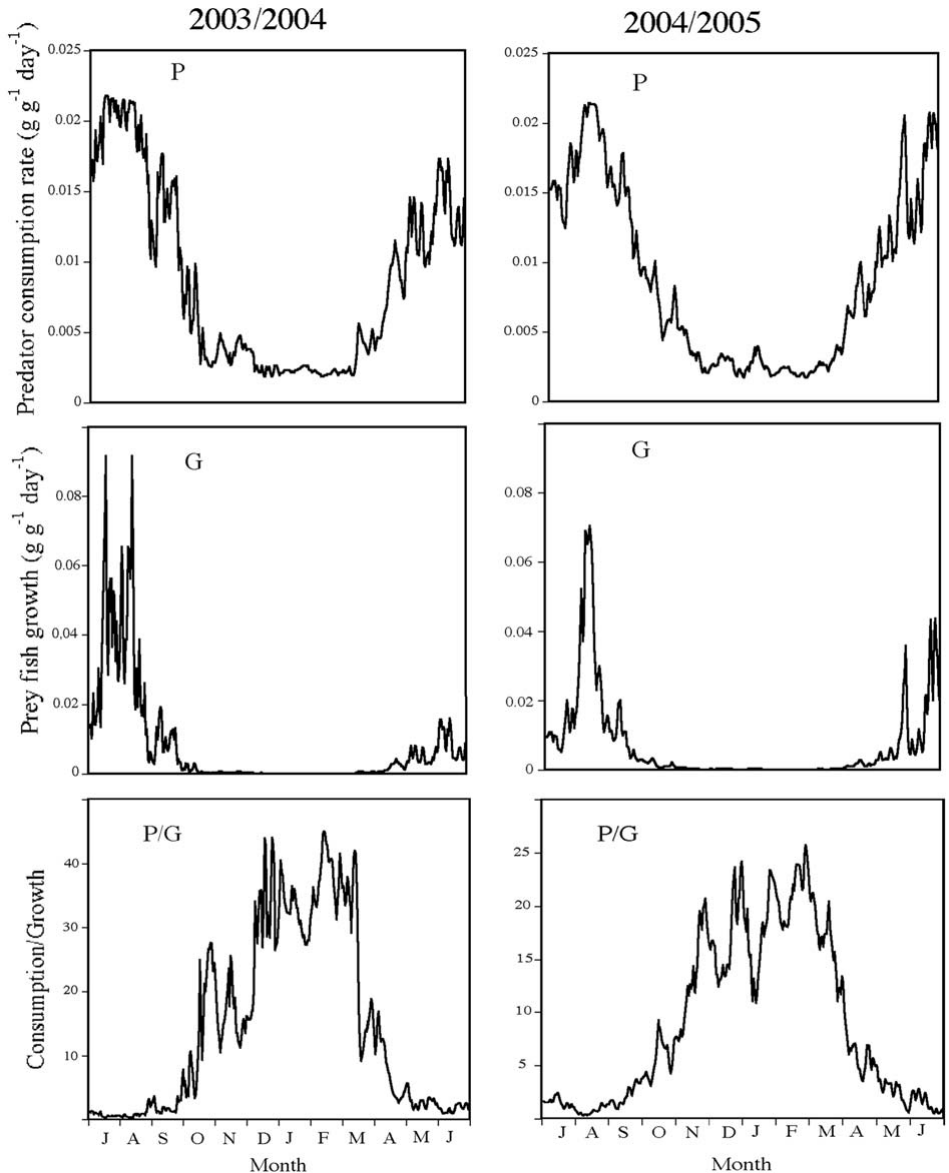
Seasonal development of piscivore consumption rate, growth rate of roach and the ratio between piscivore consumption and roach growth in Lake Krankesjön during 2003/4 and 2004/5.

In the conceptual model, we hypothesize that the consumption rates of predators, and thus mortality rates for prey fish, should be highest during summer and decrease to low levels during winter (Fig. 1). The bioenergetics modelling confirmed this; although pike consumption rates decreased up to 90% during winter the modelling results indicate that consumption rate of pike is always above zero (Fig. 2). In prey fish, the potential growth rate is also highest during summer when the production of food (zooplankton and other invertebrates) is highest, but very low when temperatures decline below 10°C (Fig. 1, 2). The lower temperature threshold for feeding and growth in roach lies in the range 12–15°C [30], [31] and the low availability of zooplankton during winter in Lake Krankesjön [30] further emphasizes the low benefits of the lake as a foraging habitat for planktivorous prey fish during this period.

To evaluate the seasonal patterns of the trade-off between predation mortality (P) and growth (G) for prey fish in the lake habitat we calculated the ratio between predator consumption rate and prey fish growth obtained from the bioenergetics modelling. The calculated trade-off varied considerably over the year, but the general patterns follow the predictions from the conceptual model, showing that the relative costs and benefits of the lake as a habitat for prey fish have a strong seasonal pattern (Fig. 2).

In the conceptual model, we also assume that the predation pressure is low in the stream all year and, that there is a constantly low growth rate for planktivorous fish (Fig. 1). Very few predators migrated from the lake to the streams and the resident predator population in the stream was very sparse. Thus, predation pressure in the streams may be considered insignificant all year. Moreover, roach mainly feed on zooplankton and the zooplankton food resource is almost absent in streams. Although zooplankton is the main food item in roach diets, macroinvertebrates may also be included to some extent [13]. However, in another study it has been found that macroinvertebrate biomass is lower in the River Silvåkrabäcken than in Lake Krankesjön (L. Ranåker, A. Nilsson & J. Brodersen, unpublished). Further, the majority of guts from roach sampled in the stream during winter were empty (J. Brodersen, unpublished). Thus, as both predation pressure and resource availability/growth is low and relatively constant throughout the year in the stream there should be little change in predation rate/growth rate ratio in the stream between seasons (Fig. 1).

**Figure 3:**
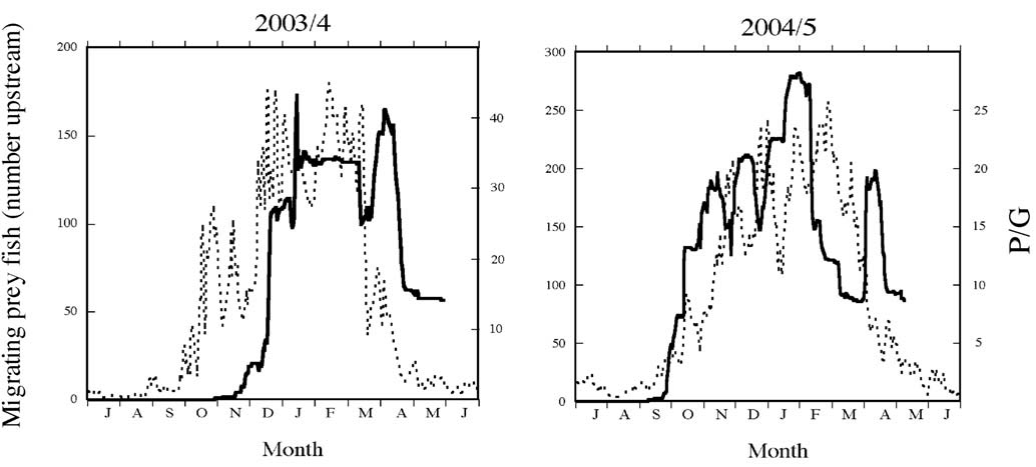
Seasonal changes in observed migration of roach (number of tagged roach in stream; black line, left y-axes) compared to predictions from seasonal changes in the piscivore consumption/roach growth ratio ((P/G; hatched line, right y-axes) during two years.

Given that there are no major seasonal changes in the P/G ratio in the stream habitat, we predicted that the stream and lake trajectories cross during periods of large changes in the lake ratio, i.e. during autumn and spring (Fig. 1, 2). When analysing the migration of individually marked fish it was clear that the general patterns of the actual migration showed a remarkable consistency with the predictions from the calculated trade-off ratios (Fig. 3). In autumn 2003 there were two dramatic increases in the calculated ratio, 24 September-16 October and 23 November -16 December and, thus, we predicted that prey fish should start to migrate in the end of September. Unfortunately, the recorders were not put in place until mid-October 2003 so we could not test this prediction, but the first recorded prey fish migrated into the stream on 21 October and migration continued until 20 December when migration intensity levelled off. This overlapped with the second period of increasing P/G ratio of the lake, i.e. was in complete agreement with the predictions. In 2004, we predicted that prey fish should start migrating during the last week of September and the observed patterns followed this prediction remarkably well (Fig. 3). The increase in the ratio in autumn 2004 showed several highs and lows and a similar pattern was found for the observed number of migrating fish which continued to increase until mid-January. In spring 2004, prey fish were predicted to migrate from the streams during the periods 14–18 March and 3–17 April when there were dramatic reductions in the P/G ratio and which closely agrees with the observed patterns (high migration out of the stream 9–20 March and 3–20 April). In 2005 the predation/growth ratio decreased gradually from 24 February to 5 April and roach started to leave the stream somewhat earlier, already on the 5 February. The calculated ratios returned to the autumn pre-migration values on 17 April 2004 and 20 April 2005 and this coincides almost exactly with the dates when the major migration down from the stream ended for the season (16 April 2004 and 17 April 2005). Thus, the start and end of the migration period were in extremely good agreement with predictions from what is expected if prey fish trade off growth gains against predation costs. Further, much of the smaller scale patterns during the winter follows predictions from changes in calculated predation/growth, suggesting that prey fish were very sensitive to changes in relative costs and gains.

An alternative explanation for the seasonal migration patterns is that roach escape low oxygen levels in the lake during winter by moving up into the tributaries. Shallow, eutrophic lakes with an extensive cover of submerged macrophytes may experience dramatic reductions in oxygen concentrations as macrophytes are decomposing during winter, sometimes down to critical levels that result in fish kills. This is especially pronounced under periods of ice- and snow-cover. Other studies have indeed explained habitat shift behaviours and migration in fish as an adaptation to avoid hypoxic conditions during winter [32], [33]. However, monitoring showed that oxygen concentrations in the lake during winter were always higher than the concentrations that limit fish performance (>7 mg/l) [34] and, in fact, always higher in the lake than in the streams, even during periods with ice cover (lake: 15.7±2.60, stream: 9.08±1.54 mg/l; mean±SD; n = 5, t = 4.92, p = 0.001). Another potential explanation is that prey fish show thermoregulatory behaviour and choose the warmest water available. The temperature was 1–2°C higher in the stream during a few winter weeks, but it was very cold in both systems then (2–4°C), i.e. well below the temperature when roach cease to feed and grow [30], [31]. Further, when averaging over the whole migration period there was no significant difference in temperature between the stream and the lake (p>0.05). Thus, neither thermoregulatory behaviour nor oxygen deficiency could explain the migration patterns of prey fish in this system.

Due to the laborious logistics associated with individual marking of fish we have focused our study on one target lake; Lake Krankesjön, southern Sweden. However, we have recorded regular mass-migrations of prey fish (mainly cyprinids) in numerous lakes in the vicinity of our study site, in other regions of Sweden and also in Denmark. Further, seasonal migrations between habitats have also been recorded by other researcher [9], [25], [35] and this suggests that mass-migration of prey fish from lakes during winter is a general and common phenomenon.

In conclusion, we have shown that prey fish undertake seasonal migrations where they leave the lake and migrate up into streams and connected wetlands during winter. Moreover, in a conceptual model we have, for the first time, provided a mechanistic explanation for mass–migration in freshwater fish. By estimating mortality and growth rates using bioenergetics modelling we were able to predict the timing of the migration with surprisingly high accuracy. Temperature affects metabolic rates and growth of fish and is further correlated to food availability and is thus the driving force behind the seasonal changes in the cost/benefit trade-off. This ultimately affects the decisions an individual fish makes with regard to habitat choice in order to maximize lifetime fitness and suggests that the large-scale migration pattern shown here has evolved in response to seasonally fluctuating trade-offs between predator avoidance and foraging gains. The result of the migration is that prey fish actually spend the major part of the year away from the lake and this could have considerable consequences for the structure and dynamics of lake ecosystems through direct and indirect food-web interactions [29], [36].

## Materials and Methods

### Study system

The study was conducted in Lake Krankesjön, a 3.4 km^2^ shallow (mean depth 1.5 m, maximum depth 3.0 m), eutrophic lake in southern Sweden. The lake has two inlet streams, Länsmansbäcken and Silvåkrabäcken and one outlet stream, Ålabäcken. Standardized survey multi-mesh gillnet fishing has shown that the fish assemblage in the lake is dominated by roach *Rutilus rutilus* (36% of total numbers) together with perch *Perca fluviatilis* (25%), white bream *Blicca bjoerkna* (12%), rudd *Scardinius erythrophthalmus* (11%) and bleak *Alburnus alburnus* (7%) (M. Svensson, unpublished). Northern pike *Esox lucius* is the dominant piscivore in the lake, together with larger size-classes of perch. The density of pike is high in Lake Krankesjön (up to 200 ind. ha^−1^, [37]).

### Oxygen and temperature

Oxygen was measured biweekly during winter months (November-March) in the lake and in the streams with an Oxyguard® oxygen probe. Temperature was recorded continuously starting on 24 October 2003 with temperature loggers (Onset Stowaway® Tidbit®) placed in the lake and in each of the inlet streams. Temperature was recorded once every three hours but in the analyses we use daily average temperature.

### Fish marking

Between September and November 2003 we caught 701 roach, 145 northern pike and 216 perch by electro-fishing in Lake Krankesjön. During the same period in 2004 we caught 592 roach, 143 northern pike and 50 perch. After being anesthetized the body weight (g) and total lengths (mm) of each fish were measured. They were then tagged with a TIRIS^®^ Passive Integrated Transponder-tag (PIT-tag) (Texas Instruments, RI-TRP-RRHP, half duplex, 134 kHz, 23.1 mm long, 3.85 mm diameter, 0.6 g (air)). All of the tagged fish were >12 cm total length. After tagging and recovery from anaesthesia, fish were released into the lake. An evaluation of PIT-tag marking techniques showed that this method results in very low mortality and no negative effects on condition [38]. The study complies with the current laws in Sweden; ethical concerns on care and use of experimental animals were followed under permission (M14-04) from the Malmö/Lund Ethical Committee.

### Fish migration

Migration of fish between the lake and the in- and outlets was monitored by passive bio-telemetry using a modified PIT-tag system originally designed for monitoring fish movement in fishways [39]. Each fish was marked with a PIT-tag that emits a signal when the fish swim through a recording antenna. The antenna was connected to a recording system that recorded the PIT-tag signal and stored it on a memory card that was exchanged every fourth day from mid-October 2003 to the end of May 2005. The recording frequency was 5 energize/receive cycles s^−1^. Two loop-shaped antennas, each covering the entire cross-section of the stream, were placed 4–6 m from each other in each of the two inlet streams and the outlet stream. By having two antennas placed close to each other we were able to determine both timing and direction of migration of individual fish . The antennas were placed 500–600 meter upstream from the lake in the two inlets and 260 meter downstream from the lake in the outlet to ensure that registered fish were migrants, i.e. not just moving in and out of the lake at the stream mouth.

### Fish in streams

Predation pressure in the stream during winter may be due to predation from both resident and migratory piscivores. In order quantify the presence of piscivores we sampled the tributaries and connected wetlands during winter by repeated electro-fishing and fykenet fishing (13 occasions).

### Bioenergetics modelling

In this study we use piscivore predation rate as a proxy for the predation risk experienced by prey fish. To estimate seasonal changes in predation rate by piscivores we used a bioenergetics model [40] that has been parameterized for northern pike, the dominant predator in the lake. Perch may also become piscivorous in lake Krankesjön. However, our tagging method limited us to study the migration pattern of roach >12 cm and prey of this size has reached a size-refuge from predation by perch in Lake Krankesjön. Thus, perch were not included in the calculation of piscivore consumption rates. Study-specific parameters entered into the model were seasonal growth rates of pike (initial size: 1040 g, yearly growth: 215 g; A. Nilsson, unpublished data from Lake Krankesjön), temperature (daily average temperature from Lake Krankesjön) and diet composition (100% fish diet). We chose to enter parameters for a specific piscivore size into the model although size structure of the piscivore population should affect the predation pressure on prey fish. However, in this study our main aim was to investigate the effects of seasonal changes in predation pressure. Due to size-constraints of the fish marking method we only marked roach larger than 120 mm. Only pike larger than 200–300 mm feed on roach of these sizes [41] and we assume that there are no major seasonal changes in the density of these size classes of pike in Lake Krankesjön. In temperate lakes, temperature should be more important for determining seasonal changes in piscivore predation pressure, and, thus, we did not take size structure and cohort strength into consideration in the bioenergetical modelling of piscivore consumption. Seasonal changes in prey fish growth rate (G) were estimated by calculating specific growth rate (g g^−1^ day^−1^) for the dominant prey fish, roach, using an empirical relationship for temperature-dependent growth in roach (g = 0.006×10^0.128T^, g = growth, T = temperature; data from [28]).
